# Synthesis and *in Vitro* Screening of Phenylbipyridinylpyrazole Derivatives as Potential Antiproliferative Agents

**DOI:** 10.3390/molecules20011031

**Published:** 2015-01-09

**Authors:** Mohammad M. Al-Sanea, Ahmed Elkamhawy, Ahmed Zakaria, Byung Sun Park, Youngjoo Kwon, So Ha Lee, Sang Woo Lee, In Tae Kim

**Affiliations:** 1 Chemical Kinomics Research Center, Korea Institute of Science and Technology, Hwarangno 14-gil 5, Seongbuk-gu, Seoul 136-791, Korea; E-Mails: a.elkamhawy@gmail.com (A.E.); aznagi2003@gmail.com (A.Z.); yangwenli1@nate.com (B.S.P); lsh6211@kist.re.kr (S.H.L.); 2 Department of Biological Chemistry, Korea University of Science and Technology, 113 Gwahangno, Yuseong-gu, Daejeon 305-333, Korea; 3 College of Pharmacy and Graduate School of Pharmaceutical Sciences, Ewha Womans University, Seoul 120-750, Korea; E-Mail: ykwon@ewha.ac.kr; 4 Department of Chemistry, Kwangwoon University, Seoul 139-701, Korea; E-Mails: lsw1018@kw.ac.kr (S.W.L.); itkim@kw.ac.kr (I.T.K.)

**Keywords:** cancer, cell lines, phenylbipyridinylpyrazole, antiproliferative agents, COMPARE analysis

## Abstract

A series of phenylbipyridinylpyrazoles was synthesized through the reaction of 2-(4-(2-chloropyridin-4-yl)-3-(3-methoxy-5-methylphenyl)-*1H-*pyrazol-1-yl)acetonitrile (**4**) with different 6-substituted pyridine-3-ylboronic acids. The final compounds **5a**–**j** were screened at 10 µM against over 60 tumor cell lines at the U.S. National Cancer Institute (NCI). In light of the NCI results, compounds **5c** and **5h** showed a broad spectrum of activity against NCI cell lines with mean growth of 53% and 58%, respectively. Compound **5e** behaved differently as it showed high degree of selectivity and potency by inhibiting 96% of growth of leukemia SR cell line at 10 µM. Standard COMPARE analyses were performed at the GI_50_ level and the results exhibit high correlation in the form of pairwise correlation coefficient (PCC) of more than 0.6 between three of the current compounds and three standard known anticancer agents. Compound **5e** demonstrated high correlation levels with merbarone (NSC S336628) with a PCC value of 0.631. Compound **5h** showed a considerably high PCC value of 0.626 with dichloroallyl lawsone, while compound **5i**, showed PCC values of 0.601 and 0.604 with both dichloroallyl lawsone and *N,N*-dibenzyldaunomycin (NSC S268242), respectively. These three standard agents have anticancer activity via two major mechanism of actions, inhibition of topoisomerase II and inhibition of biosynthesis of pyrimidine nucleotides, therefore, compounds **5a**–**j** are promising therapeutic agents for targeting different human malignancies. Prediction of drug-likeness and toxicity of these newly synthesized derivatives were also considered.

## 1. Introduction

Cancer has been the leading worldwide cause of death, and accounted for 7.6 million deaths in 2008 according to WHO reports [1]. The conventional cancer treatments such as surgery, radiation and cytotoxic chemotherapy seem to be no longer efficient neither for the complete abolition of disease nor for the improvement of cancer patients’ quality of life. This has driven the development of a variety of new anticancer agents with more potent, high specific and low cytotoxic properties.

The term anti-metabolite is used to cover a number of cytotoxic agents that act by masquerading as purines or pyrimidines, the building blocks of DNA. Inhibition of biosynthesis of pyrimidine nucleotides has become a new strategy in cancer treatment. Dichlorallyl lawsone [2-hydroxy-3-(3,3-dichloroallyl)-1,4-naphthoquinone] is of interest as an cytotoxic agent through inhibition of biosynthesis of pyrimidine nucleotides. It stimulates the incorporation of [^14^C]uridine into macromolecules, decreases the pool sizes of uridine triphosphate, prevents the pyrazofurin-induced accumulation of orotidine and orotic acid, and exerts cytotoxicity that could be prevented or reversed almost completely by uridine [2].

Another cancer treatment strategy is the inhibition of DNA-topoisomerases. This enzyme are omnipresent nuclear enzymes which induce transient breaks in the DNA allowing DNA strands or double helices to pass through each other and play essential roles in DNA replication and transcription [3,4]. DNA topoisomerases fall into two major classes: the type I enzymes that induce single stranded cuts in DNA, and the type II enzymes that cut and pass double stranded DNA [5,6]. Therefore, tumor cell multiplication and growth can be controlled by interfering with topoisomerases and then tumor control could be achievable [7,8]. In particular, different compounds with a pyrazole core show an interesting antiproliferative activity [9,10,11,12].

Herein, a new series of phenylbipyridinylpyrazole scaffold-based compounds has been synthesized and biologically screened against over 60 cancerous cell lines in order to check their* in vitro* anti-proliferative activity. In order to gain insight into the mechanism of the cytotoxic action of some final compounds, matching mean graph fingerprint (Compare Analysis) has been applied. The Compare Analysis results have suggested two possible mechanisms of action for these phenyl-bipyridinylpyrazole scaffold-based compounds: (i) topoiomerase IIα inhibition and (ii) pyrimidine nucleotide synthesis inhibition.

## 2. Results and Discussion

### 2.1. Chemistry

**Scheme 1:**
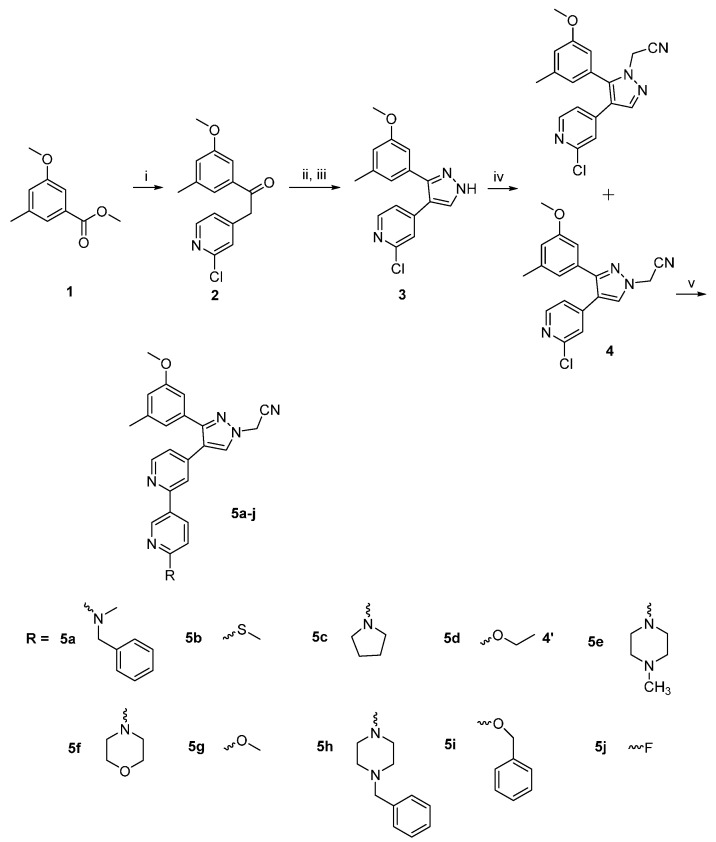
Synthesis of target compounds: (i) LHMDS, 2-chloro-4-methylpyrimidine, THF, N_2_, rt, 18 h, 85%; (ii) DMF-DMA, 90 °C, 12 h; (iii) hydrazine hydrate, abs. ethanol, rt, 2 h, 80%; (iv) K_2_CO_3_, iodoacetonitrile, acetone, reflux, 4 h, 90%; (v) 6-substituted pyridine-3-boronic acid, Pd(PPh_3_)_2_Cl_2_, K_2_CO_3_, N_2_, CH_3_CN/H_2_O (4:1), 78 °C, 4 h.

The preparation of bipyridinylpyrazole compounds **5a**–**j** is outlined in Scheme 1. Substituted benzoic acid ester **1** is subjected to a nucleophilic attack on its trigonal carbonyl carbon by an activated methylene group of 2-chloro-4-methylpyrimidine as reported in the literature [13,14,15].

The methylene activation was achieved by dropwise addition of lithium hexamethyldisilazide (LHMDS) in dry THF at room temperature. The resulting tautomeric α,β-unsaturated ketone **2** was then converted into the pyrazole derivative **3** through two sequential steps. First compound **2** was heated with an excess of *N,N*-dimethylformamide dimethylacetal for 12 h, and the resulting product was then cyclized into the pyrazole derivative **3** with hydrazine monohydrate in absolute ethanol. The reaction of the resulting pyrazole **3** with iodoacetonitrile in the presence of excess potassium carbonate produced two regioisomers; isomer **4** having a lower R_f_ value of 0.84 (ethyl acetate/hexane 1:1, v/v) and another isomer having a higher R_f_ value of 0.74 [13]. The location of the acetonitrile moiety on the pyrazole nitrogens was assigned and confirmed using 2D-NOESY NMR as addressed before in the literature [16]. The two isomers were separated chromatographically using a mixed solvent of ethyl acetate and hexane (1:1, v/v) and isomer **4** is taken to the next Suzuki coupling step with a series of 4-substituted pyridineboronic acids to furnish final compounds **5a**–**j**.

### 2.2. Biological Evaluation

#### 2.2.1.* In Vitro* Anticancer Screening

Compounds **5a**–**j** were selected for evaluation of their antiproliferative activity by the National Cancer Institute (NCI, Bethesda, MD, USA), on the basis of their degree of structural variation and computer modeling techniques. The compounds were subjected to* in vitro* anticancer assays against tumor cells in a full panel of 60 different human tumor cell lines representing nine different tissues (leukemia, melanoma and cancers of the lung, colon, brain, ovary, breast, prostate, and kidney). The compounds were tested at a single dose concentration of 10 µM and the cultures were incubated for 48 h. The results for each compound are reported as a mean graph of the percent growth of the treated cells when compared to the untreated control cells. The percentage growth of the tested compounds against the full 60-cell lines panel is illustrated in Table 1.

**Figure 1:**
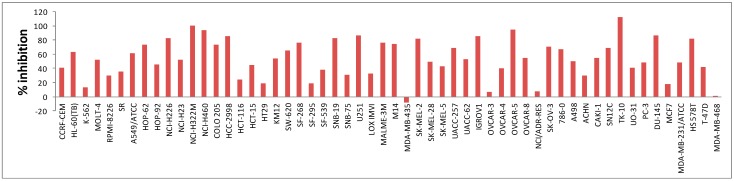
Inhibitory effects of compound **5c** at 10 uM concentration over 60 cell lines.

The mean growth percentages of all of the tested compounds over full 60-cell lines are illustrated in Table 2. Compounds **5c** and **5h** exhibited the lowest mean percentage growth against the full 60-cell line panel, which means they had a broad spectrum of activity against different cancer cell lines. It is worth mentioning that compound **5c** showed strong inhibitory activity (>90%) against three cell lines (OVCAR-3, NCI/ADR-RES, MDA-MB-468) with a lethal effect rather than growth inhibition on MDA-MB-435 melanoma cell line (this appears as a negative column as shown in Figure 1).

**Figure 2:**
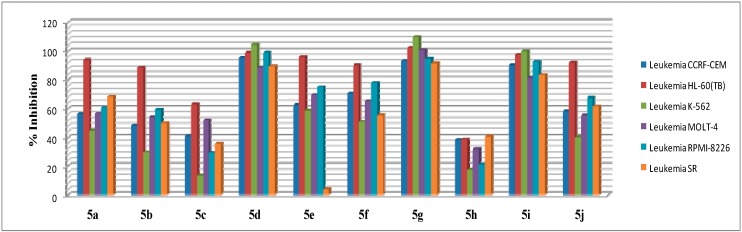
Inhibitory effects of the tested target compounds at 10 μM concentration over all six tested leukemia cell lines.

Compound **5h**, in particular, exhibited considerable efficacy against all leukemia cell lines with a percentage growth range of 17%–40% (Figure 2). Regarding the sensitivity against individual cell lines, compound **5e** showed observed single strong cell growth inhibition of one leukemia cell line, SR, with a growth percentage of 4.04%. As the mean percentage growth of Compound **5e** is 85%, high degree of selectivity profile has become obvious and it could be useful in targeting specific leukemia SR cell line.

By referring to the results of the inhibitory effects of the tested compounds summarized in Table 1 and Table 2, and correlating between the structural variations and the resulted anticancer activities, we cannot establish precise structure activity relationships (SAR) between the bipyridinylpyrazole scaffold and the current NCI screening results. However, some clues for understanding the currently available screening results could be deduced, which perhaps could be useful for our future work in developing this scaffold. Generally, substitution at the terminal pyridinyl group with a hydrophobic bulky moiety is more favorable than small groups like methoxy or ethoxy. Deep hydrophobic interaction with the complementary hydrophobic pocket at the target macromolecule(s) could be achieved if 4-pyridinyl position has been substituted with a phenyl moiety carried on a spacer like piperazine or oxymethylene bridge groups, as revealed by compounds **5h** and **5i**.

**Table 1:** Percentage of cell growth for the tested compounds against 60 tumor cell lines.

Cell Lines		Percentage of Cell Growth ^a^									
		5a	5b	5c	5d	5e	5f	5g	5h	5i	5j
**Leukemia**	CCRF-CEM	56.06	48.23	40.94	95.08	62.47	70.45	92.95	38.31	90.4	57.97
	HL-60(TB)	93.71	88.74	63	98.85	95.75	90.3	102.6	38.58	97.47	91.84
	K-562	44.4	29.76	13.4	104.4	58.46	50.75	109.7	17.62	99.8	40.04
	MOLT-4	56.34	54.13	51.91	88.68	69.09	65.33	100.7	31.55	81.16	55.14
	RPMI-8226	60.48	58.94	29.52	98.94	75.11	77.93	94.57	20.91	92.35	67.43
	SR	67.87	49.8	35.47	89.52	4.04	55.17	91.36	40.73	83.31	61.59
**NSCLC**	A549/ATCC	87.9	91.94	61.47	85.83	102.7	87.01	86.18	60.74	88.37	74.32
	HOP-62	107.0	95.83	73.86	85.9	104.2	88.22	99.97	110.8	87.28	110.1
	HOP-92	69.97	43.6	45.34	49.16	44.52	46.04	52.65	52.61	60.87	84.65
	NCI-H226	88.11	74.6	83.09	87.54	92.64	88.58	86.04	74.88	92.03	81.26
	NCI-H23	85.56	83.56	51.85	99.59	94.98	92.09	96.39	72.15	92.21	86.9
	NCI-H322M	91.42	77.33	100.2	113.6	89.82	85.69	101.0	95.42	93.24	97.23
	NCI-H460	94.42	98.84	94.09	102.9	102.4	101.6	96.78	57.17	98.8	94.88
**Colon Cancer**	COLO 205	82.6	76.8	73.72	96.46	79.75	100.9	100.4	41.02	102.3	82.76
	HCC-2998	88.15	84.24	85.57	98.48	100.0	99.21	106.2	67.09	99.95	92.82
	HCT-116	67.45	60.72	24.5	91.03	61.7	86.09	103.9	43.2	85.8	69.84
	HCT-15	57.32	52.74	44.88	94.81	70.42	68.16	93.73	24.6	88.06	53.49
	HT29	37.1	48.01	19.08	104.3	51.26	77.25	94.72	11.94	92.24	37.66
	KM12	86.66	54.28	54.32	104.7	95.36	79.26	102.7	43.97	100.5	87.17
	SW-620	92.45	76.97	65.02	102.5	82.84	94.36	92.91	60.55	97.48	77.97
**CNS Cancer**	SF-268	100.2	98.85	76.61	100.6	104.1	94.35	96.35	76.17	90.2	96.09
	SF-295	89.92	84.01	18.24	88.62	95.43	56.53	95.6	63.38	97.89	90.11
	SF-539	95.97	78.11	37.87	95.06	88.77	79.27	96.35	30.21	92.94	88.07
	SNB-19	92.1	85.25	82.27	91.29	89.56	90.84	92.17	79.89	94.61	88.35
	SNB-75	95.38	44.53	30.49	66.83	55.01	63.06	76.9	65.89	73.17	85.68
	U251	94.47	91.11	86.66	93.01	95.17	96.88	93.53	68.64	93.18	88.99
**Melanoma**	LOX IMVI	69.37	61.68	32.53	97.91	89.63	65.72	97.2	32.95	91.89	67.44
	MALME-3M	107.5	77.19	76.41	102.9	93.51	92.92	104.1	79.55	97.85	104.28
	M14	90.13	92	74.58	93.6	107.8	98.58	104.3	64.79	120.8	100.25
	MDA-MB-435	96.21	46.59	−8.52	96.15	96.69	59.14	102.0	68.22	106.4	95.83
	SK-MEL-2	105.3	117.9	82.05	121.0	123.4	96.91	101.4	83.3	120.9	112.4
	SK-MEL-28	101.5	90.11	48.94	94.86	97.45	86.64	108.0	69.36	102.6	107.17
	SK-MEL-5	93.55	77.97	42.91	97.01	81.82	73.07	96.02	70.71	100.4	93.82
	UACC-257	107.7	114.0	69.05	97.1	114.1	95.97	105.4	83.45	102.2	105.51
	UACC-62	73.42	72.26	52.73	87.66	94.86	80.73	87.72	65.51	85.59	87.91
	IGROV1	109.7	69.24	85.16	110.2	105.7	93.48	95.63	87.36	101.5	96.37
**Ovarian Cancer**	OVCAR-3	98.29	77.61	6.29	104.3	97.5	92.24	105.0	55.41	94.35	91.6
	OVCAR-4	87.46	63.79	39.58	90.49	82.78	81.73	100.8	60.1	90.31	90.57
	OVCAR-5	91.13	72.62	94.75	87.78	91.08	90.84	84.93	77.7	74.9	91.76
	OVCAR-8	81.22	84.54	55.05	89.33	92.19	84.12	88.68	63.03	89.73	79.8
	NCI/ADR-RES	83.72	77.68	7.25	92.72	91.54	66.54	95.56	60.92	84.09	75.16
	SK-OV-3	98.39	91.79	70.56	95.22	106.3	101.1	104.1	81.49	98.24	92.76
**Renal Cancer**	786-0	91.57	76.97	67.03	91.19	81.84	86.41	98.75	67.64	92	83.91
	A498	91.0	110.2	50.46	103.4	80.4	93.82	97.71	87.81	87.49	98.66
	ACHN	82.25	63.48	29.86	87.6	81.27	55.86	91.97	51.64	75.87	95.16
	CAKI-1	82.27	72.03	54.56	83.76	90.18	84.89	87.76	61.49	86.4	81.52
	SN12C	78.84	72.33	68.88	90.66	78.88	85.35	90.12	51.19	87.72	79.58
	TK-10	109.8	100.5	112.1	107.4	113.3	115.5	96	90.95	86.43	111.5
	UO-31	91.95	61.17	41.05	82.51	67.72	71.4	70.5	57.74	69.35	80.58
**Prostate Cancer**	PC-3	61.14	65.35	48.35	85.21	87.85	67.24	83.77	34.01	86.85	69.44
	DU-145	93.7	84.57	86.53	112.5	90.3	96.15	109.2	66.92	111.1	93.49
**Breast Cancer**	MCF7	76.85	26.85	18.15	98.85	76.91	43.18	102.5	28.28	89.1	63.72
	MDA-MB-231/ATCC	60.44	52.64	48	89.4	79.16	69.26	80.57	31.94	77.26	47.6
	HS 578T	84.41	81.85	82.21	109.4	91.78	89.77	85.33	65.96	82.47	79.81
	T-47D	57.58	26.9	42.13	70.07	72.63	72.37	80.58	28.42	68.23	40.82
	MDA-MB-468	68.1	19.33	1.31	87.94	63.44	76.26	86.7	40.28	69.27	61.8

Note: ^a^ screened at 10 µM concentration.

**Table 2:** Screening data of the tested compounds with the most sensitive cell lines and mean percentage growth.

Cell Lines	5a	5b	5c	5d	5e	5f	5g	5h	5i	5j
**K-562**	- ^a^	29.76	13.4	-	-	-	-	17.62	-	-
**SR**	-	-	35.47	-	4.04	-	-	-	-	-
**HT29**	37.1	-	19.08	-	-	-	-	11.94	-	37.66
**MDA-MB-435**	-	-	−8.52	-	-	-	-	-	-	-
**OVCAR-3**	-	-	6.29	-	-	-	-	-	-	-
**NCI/ADR-RES**	-	-	7.25	-	-	-	-	-	-	-
**MCF7**	-	26.85	18.15	-	-	-	-	28.28	-	-
**MDA-MB-468**	-	19.33	1.31	-	-	-	-	-	-	-
**Mean % growth**	84	72	53	94	85	81	94	58	82	91

Note: ^a^ Values which exceed 40% growth.

COMPARE analyses was applied to all the final compounds in order to investigate the similarity of their cytotoxicity patterns (mean graph fingerprints) with data from known anticancer standard agents, NCI active synthetic compounds and natural extracts, which are available in public available databases. Such an analysis is based on comparing the differential growth inhibition patterns for cultured cell lines and can potentially provide insight into the mechanism of the cytotoxic action. If the data pattern correlates well with those of compounds found in a standard agent database (pairwise correlation coefficient (PCC > 0.6), the compound of interest may have the same mechanism of action [17,18]. On the other hand, if the activity pattern does not correlate with any standard agent, it is possible that the compound has a novel mechanism of action. Standard COMPARE analyses were performed at the GI_50_ level.

The results of that screening exhibit high correlation (PCC > 0.6) between three of our compounds and three known anticancer standard agents. Compound **5e** demonstrated high correlation levels with merbarone (NSC S336628) with a PCC value of 0.631. Compound **5h** showed a considerably high PCC value of 0.626 with dichloroallyl lawsone (NSC S126771), while compound **5i** had PCC values of 0.601 and 0.604 with both dichloroallyl lawsone and *N,N*-dibenzyldaunomycin (NSC S268242), respectively. Such similarity in COMPARE results could indicate a resemblance in the corresponding mechanisms of action. Merbarone and *N,N*-dibenzyldaunomycin share the same mechanism of action as anti-cancer agents by inhibiting the topoisomerase II enzyme. The DNA-topoisomerases are omnipresent nuclear enzymes that play vital roles in DNA metabolism events, such as replication and transcription. Because of its critical role in the maintenance and replication of DNA during proliferation, cells become highly vulnerable when these functions are disrupted [7,8].

#### 2.2.2. Topoisomerase IIα Inhibitory Activity

From the topoisomerase IIα screening results, we found that compounds **5e** and **5i**, with inhibitory activity of 1.2%, 0% and 0% did not show topoisomerase IIα inhibitory activity at three different concentrations, 100, 25 and 5 μM (as compared to 78.8%, 50.3% and 15.0% of etoposide at 100, 25 and 5 μM, respectively).

**Figure 3:**
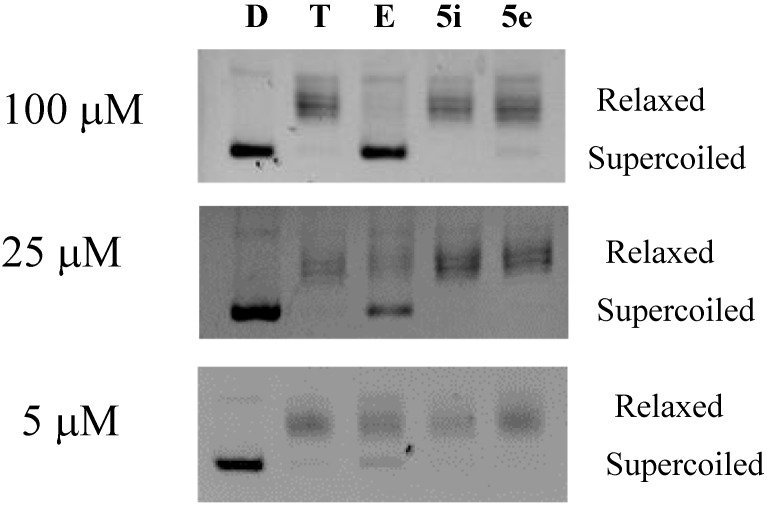
Topoisomerase IIα inhibitory activities of the compounds, 5e and 5i. The compounds were examined in a final concentration of 5, 25 and 100 µM, respectively, as designated. Lane D: pBR322 only, Lane T: pBR322 + topoisomerase IIα, Lane E: pBR322 + topoisomerase IIα + etoposide, Lane 5e: pBR322 + topoisomerase IIα + compound **5e** in designated concentrations, Lane 5i: pBR322 + topoisomerase IIα + compound **5i** in designated concentrations.

Figure 3 shows the results of topoisomerase IIα inhibitory activities of the two evaluated compounds **5e** and **5i**. It means that the mechanism of action of these two promising antiproliferative compounds is not topoisomerase IIα inhibition as suggested by the Compare Analysis correlation study.

### 2.3. Assessment of Toxicity Profiles and Lipinski’s Rule of Five

The OSIRIS program is generally used for prediction of various drug-relevant properties and overall toxicity and some undesired effects like mutagenicity or a poor intestinal absorption. Herein, OSIRIS was applied for predicting those issues for our designed compounds. None of the target compounds showed any predicted toxicity risks.

The total polar surface area (TPSA) was calculated as it is a key property that has been linked to drug bioavailability. Thus, passively absorbed molecules with a TPSA > 140 are thought to have low oral bioavailability [19]. As all the final compounds have TPSA value ranging from 76 to 89 (Table 3), they hypothetically should show good passive oral absorption. Furthermore, the Lipinski’s rule of five was reviewed as it clearly estimates the ADME properties which are considered important in the drug development stage and clinical trials. Predictions of ADME properties for the studied compounds are given in Table 3. The results showed that most of the target compounds obey these rules suggesting that the synthesized compounds would be possible drug molecules.

**Table 3:** Total polar surface area, calculated Lipinski’s rule of five, and toxicity risks for final compounds.

Comp No.	TPSA ^a^	MW ^b^	nHBA ^c^	nHBD ^d^	V ^e^	RB ^f^	Volume ^g^	Clog P ^h^	Toxicity ^i^
**5a**	85.87	425.492	7	0	0	7	389.283	3.34	-^j^
**5b**	83.112	479.588	8	0	0	6	445.429	2.78	-
**5c**	89.108	466.545	8	0	0	6	425.069	2.68	-
**5d**	85.87	411.465	7	0	0	6	372.481	2.94	-
**5e**	83.112	555.686	8	0	2	8	517.079	4.2	-
**5f**	76.636	427.533	6	0	0	6	381.625	3.23	-
**5g**	79.874	450.546	7	0	0	6	416.084	3.5	-
**5h**	79.874	500.606	7	0	2	8	464.491	4.32	-
**5i**	76.636	399.429	6	0	0	5	351.867	3.11	-
**5j**	85.87	487.563	7	0	1	8	444.13	4.35	-

Notes: ^a^ Total polar surface area; ^b^ Molecular weight; ^c^ Number of hydrogen bond acceptor; ^d^ Number of hydrogen bond acceptor; ^e^ Number of violation to Lipinski’s rule of five; ^f^ Rotatable bonds; ^g^ molecular volume; ^h^ calculated lipophilicity; ^i^ no toxicity risks (mutagenicity, tumorigenicity, irritancy, reproductive effects).

## 3. Experimental Section

### 3.1. Chemistry

#### 3.1.1. General

Commercially available reagents and anhydrous solvents were commercially available and used without further purification. Thin layer chromatography (TLC) analyses were performed with precoated silica gel 60 F254 plates. NMR spectra were recorded with a Bruker 300 MHz spectrometer at ambient temperature with the residual solvent peaks as internal standards. The line positions of multiplets were given in ppm (δ) and the coupling constants (*J*) were given in hertz. The Liquid Chromatography high-resolution mass spectra (LC-HRMS, electron spray ionization) experiments were performed with a Synapt G2 Q-TOF MS (Waters MS Technology, Manchester, UK) mass analyzer. Data were acquired in the positive ion mode. Calibration was performed with an external calibration mixture immediately prior to analysis. Melting points were taken on a Thomas-Hoover Capillary Melting Apparatus and were uncorrected. Flash chromatography was performed on columns of Merck silica gel (230–400 mesh, 40–63 μm) with ethyl acetate/hexane or methanol/dichloromethane as eluents. The synthesis and analytical data of compounds **2**, **3** and **4** were published in the literature [13].

#### 3.1.2. General Procedure for Synthesis of Compounds **5a**–**j**

A mixture of compound **4** (100 mg, 0.29 mmol), aryl boronic acid (0.35 mmol), bis(triphenylphosphine) palladium(II) dichloride (9 mg, 0.013 mmol) and K_2_CO_3_ (71 mg, 0.52 mmol) was placed in a mixed solvent of THF and water (4:1, v/v, 10 mL). N_2_ gas was bubbled into this mixture for 10 min, and then the mixture was heated at 70 °C while stirring under N_2_ for 12 h. The reaction mixture was left to cool at room temperature, and extracted with ethyl acetate (100 mL × 3). The combined organic extracts were dried over anhydrous MgSO_4_ and evaporated under vacuum. The target compounds **5a**–**j** were separated in pure form by column chromatography (silica gel) using the proper ratio of ethyl acetate and *n*-hexane.

*2-(3-(3-Methoxy-5-methylphenyl)-4-(2-(6-(benzyl(methyl)amino)pyridin-3-yl)pyridin-4-yl)-1H-pyrazole-1-yl)acetonitrile* (**5a**). Flash column chromatography was carried out using hexane/ethyl acetate, 1:1 (v/v) to give the title compound as a yellow solid. Yield: 65%. mp 59–61 °C. ^1^H-NMR (CDCl_3_) δ 2.23 (s, 3H), 3.05 (s, 3H), 3.62 (s, 3H), 4.79 (s, 2H), 5.08 (s, 2H), 6.51 (d, *J =* 8.96 Hz, 1H), 6.68 (s, 1H), 6.73 (s, 1H), 6.86 (s, 1H), 6.97 (d, *J =* 5.08 Hz, 1H), 7.29 (m, 5 H), 7.47 (s, 1H), 7.73 (s, 1H), 7.98 (dd, *J =* 8.96, 2.08 Hz, 1H), 8.45 (d, *J =* 5.08, 1H), 8.58 (d, *J =* 2.08, 1H). ^13^C-NMR (CDCl_3_) δ 24.89, 36.41, 39.92, 53.32, 55.26, 105.63, 110.78, 113.52, 115.73, 118.16, 120.40, 120.53, 121.67, 127.11, 128.63, 130.08, 132.75, 136.14, 138.18, 139.97, 140.96, 146.59, 149.55, 151.49, 155.79, 159.70; positive ion ESI-MS *m/z* 523.2220 (M+Na)^+^.

*2-(3-(3-Methoxy-5-methylphenyl)-4-(2-(6-(methylthio)pyridin-3-yl)pyridin-4-yl)-1H-pyrazole-1-yl)acetonitrile* (**5b**). Flash column chromatography was carried out using hexane/ethyl acetate, 1:1 (v/v) to give the title compound as a white solid. Yield: 88.7%. mp 71–73 °C. ^1^H-NMR (CDCl_3_) δ 2.24 (s, 3H), 2.53 (s, 3H), 3.65 (s, 3H), 5.12 (s, 2H), 6.70 (s, 1H), 6.72 (s, 1H), 6.84 (s, 1H), 7.11 (d, *J =* 5.08 Hz, 1H), 7.19 (s, 1H), 7.54 (s, 1H), 7.82 (s, 1H), 7.99 (dd, *J =* 8.88, 2.08 Hz, 1H), 8.52 (d, *J =* 5.08 Hz, 1H), 8.78 (d, *J =* 2.08 Hz, 1H). ^13^C-NMR (CDCl_3_) δ 11.31, 19.45, 37.93, 53.24, 108.99, 111.43, 113.61, 117.87, 118.5, 119.2, 119.3, 127.48, 128.26, 130.56, 132.19, 138.08, 139.84, 145.64, 149.59, 152.41, 157.75, 159.46; positive ion ESI-MS *m/z* 450.1364 (M+Na)^+^.

*2-(3-(3-Methoxy-5-methylphenyl)-4-(2-(6-(1-pyrrolidinyl)pyridin-3-yl)pyridin-4-yl)-1H-pyrazole-1-yl)acetonitrile* (**5c**). Flash column chromatography was carried out using hexane/ethyl acetate, 1:1 (v/v) to give the title compound as a white solid. Yield: 92%. mp 123–125 °C. ^1^H-NMR (CDCl_3_) δ 1.97 (s, 4H), 2.23 (s, 3H), 3.47 (s, 4H), 3.63 (s, 3H), 5.1 (s, 2H), 6.38 (d, *J =* 8.92 Hz, 1H), 6.68 (s, 1H), 6.73 (s, 1H) , 6.87 (s, 1H), 6.96 (d, *J =* 5.00 Hz, 1H), 7.46 (s, 1H), 7.74 (s, 1H) , 8.02 (dd, *J =* 8.92, 2.12 Hz, 1H), 8.45 (d, *J =* 5.00 Hz, 1H), 8.56 (d, *J =* 2.12 Hz, 1H). ^13^C-NMR (CDCl_3_) δ 21.51, 25.51, 39.90, 47.10, 110.73, 113.49, 115.77, 117.8, 118.04, 120.61, 121.64, 122.53, 129.7, 129.9, 132.75, 136.04, 139.91, 140.73, 149.9, 151.44, 159.67; positive ion ESI-MS *m/z* 473.2060 (M+Na)^+^.

*2-(3-(3-Methoxy-5-methylphenyl)-4-(2-(6-ethoxypyridin-3-yl)pyridin-4-yl)-1H-pyrazole-1-yl)acetonitrile* (**5d**). Flash column chromatography was carried out using hexane/ethyl acetate, 1:2 (v/v) to give the title compound as a white solid. Yield: 48%. mp 58–60 °C. ^1^H-NMR (CDCl_3_) δ 1.32 (t, *J =* 6.92 Hz, 3H), 2.21 (s, 3H), 3.62 (s, 3H), 4.29 (q, *J =* 6.92 Hz, 2H), 4.81 (s, 2H), 5.21 (d, *J =* 8.96 Hz, 1H), 6.27 (s, 1H), 6.54 (s, 1H), 6.67 (s, 1H), 6.7 (s, 1H), 6.73 (s, 1H), 6.84 (s, 1H), 7.04 (s, 1H), 7.48 (s, 1H), 7.71 (s, 1H), 8.02 (d, *J =* 7.96 Hz, 1H), 8.46 (s, 1H), 8.48 (d, *J =* 7.96 Hz, 1H). ^13^C-NMR (CDCl_3_) δ 14.67, 21.51, 54.96, 55.28, 62.08, 110.91, 111.09, 115.29, 115.38, 118.80, 118.94, 119.08, 121.98, 127.99, 133.14, 137.24, 139.92, 141.48, 151.17, 155.11, 159.69, 164.46, 169.19, 172.89.

*2-(3-(3-Methoxy-5-methylphenyl)-4-(2-(6-(4-methylpiprazine-1-yl)pyridin-3-yl)pyridin-4-yl)-1H-pyrazole-1-yl)acetonitrile* (**5e**). Flash column chromatography was carried out using hexane/ethyl acetate, 1:5 (v/v) to give the title compound as a white solid. Yield: 25%. mp 84–86 °C. ^1^H-NMR (CDCl_3_) δ 2.23 (s, 4H) 2.34 (s, 3H), 2.55 (s, 4H), 3.62 (s, 3H), 5.11 (s, 2H), 6.62 (d, *J =* 8.96 Hz, 1H), 6.68 (s, 1H), 6.73 (s, 1H), 6.86 (s, 1H), 6.99 (d, *J =* 5.08 Hz, 1H), 7.46 (s, 1H), 7.74 (s, 1H), 7.98 (dd, *J =* 8.96, 2.24 Hz, 1H), 8.46 (d, *J =* 5.08 Hz, 1H), 8.56 (d, *J =* 2.24 Hz, 1H). ^13^C-NMR (CDCl_3_) δ 21.51, 39.91, 44.64, 45.82, 54.55, 55.29, 110.82, 113.57, 115.63, 115.70, 118.37, 120.49, 121.68, 124.54, 130.09, 132.79, 136.07, 139.93, 140.72, 146.74, 150.01, 151.42, 155.69, 159.22, 159.68; positive ion ESI-MS *m/z* 480.2509 (M+H)^+^.

*2-(3-(3-Methoxy-5-methylphenyl)-4-(2-(6-morpholinopyridin-3-yl)pyridin-4-yl)-1H-pyrazole-1-yl-)acetonitrile* (**5f**). Flash column chromatography was carried out using hexane/ethyl acetate, 1:5 (v/v) to give the title compound as a yellow solid. Yield: 73%. mp 85–87 °C. ^1^H-NMR (CDCl_3_) δ 2.21 (s, 3H), 3.48 (t, *J =* 4.68 Hz,4H), 3.6 (s, 3H), 3.72 (t, *J =* 4.68 Hz, 4H), 5.07 (s, 2H), 6.58 (d, *J =* 8.84 Hz, 1H), 6.66 (s, 1H) 6.72 (s, 1H), 6.85 (s, 1H), 6.97 (d, *J =* 5.04 Hz, 1H), 7.44 (s, 1H), 7.71 (s, 1H), 7.98 (dd, *J =* 8.84, 2.08, 1H), 8.45 (d, *J =* 5.04 Hz, 1H), 8.56 (d, *J =* 2.08 Hz, 1H). ^13^C-NMR (CDCl_3_) δ21.52, 39.85, 45.41, 55.18, 66.67, 106.44, 110.90, 113.76, 115.63, 118.23, 118.38, 120.26, 121.69, 124.67, 128.56, 132.06, 132.87, 136.05, 139.91, 140.87, 146.50, 151.33, 155.52, 159.51, 159.68; positive ion ESI-MS *m/z* 467.2186 (M+H)^+^.

*2-(3-(3-Methoxy-5-methylphenyl)-4-(2-(6-methoxypyridin-3-yl)pyridin-4-yl)-1H-pyrazole-1-yl)-acetonitrile* (**5g**). Flash column chromatography was carried out using hexane/ethyl acetate, 1:2 (v/v) to give the title compound as a white solid. Yield: 90.6%. mp 176–178 °C. ^1^H-NMR (CDCl_3_) δ 2.21 (s, 3H), 3.61 (t, *J =* 4.68 Hz, 4H), 3.89 (s, 3H), 4.81 (t, *J =* 4.68 Hz, 4H), 6.26 (s, 1H), 6.55 (brs, 1H), 6.67 (s, 1H), 6.69 (s, 1H), 6.72 (s, 1H), 6.84 (s, 1H), 7.03 (d, *J =* 4.8 Hz, 1H), 7.47 (s, 1H), 7.69 (s, 1H), 8.03 (d, *J =* 8.56 Hz, 1H), 8.46 (d, *J =* 4.8 Hz, 1H), 8.5 (s, 1H). ^13^C-NMR (CDCl_3_) δ 21.52, 53.45, 54.96, 55.18, 110.82, 115.30, 115.38, 115.65, 118.80, 118.95, 119.13, 121.68, 128.39, 133.15, 137.27, 139.92, 141.29, 150.04, 151.16, 155.18, 159.69, 164.66, 169.19.

*2-(3-(3-Methoxy-5-methylphenyl)-4-(2-(6-(4-benzyl-1-piprazinyl)pyridin-3-yl)pyridin-4-yl)-1H-pyrazole-1-yl)acetonitrile* (**5h**). Flash column chromatography was carried out using hexane/ethyl acetate, 1:3 (v/v) to give the title compound as a yellow solid. Yield: 70%. mp 71–73 °C. ^1^H-NMR (CDCl_3_) δ 2.23 (s, 3H), 2.52 (brs, 4H), 3.53 (s, 2H), 3.58 (s, 3H), 3.62 (s, 3H), 5.09 (s, 2H), 6.60 (d, *J =* 8.8 Hz), 6.68 (s, 1H), 6.73 (s, 1H), 6.86 (s, 1H), 6.98 (brs, 1H), 7.21 (m, 5H), 7.45 (s, 1H), 7.72 (s, 1H), 7.98 (d, *J =* 8.8 Hz, 1H), 8.46 (brs, 1H), 8.55 (s, 1H). ^13^C-NMR (CDCl_3_) δ 21.51, 39.90, 45.5, 52.72, 55.29, 63.5, 106.54, 110.74, 113.48, 115.68, 115.75, 118.18, 118.33, 120.61, 121.66, 128.39, 129.48, 129.99, 132.77, 135.99, 139.93, 140.67, 146.77, 150.04, 151.45, 155.81, 159.39, 159.69; positive ion ESI-MS *m/z* 556.2825 (M+H)^+^.

*2-(3-(3-Methoxy-5-methylphenyl)-4-(2-(6-benzyloxypyridin-3-yl)pyridin-4-yl)-1H-pyrazole-1-yl)acetonitrile* (**5i**). Flash column chromatography was carried out using hexane/ethyl acetate, 1:1 (v/v) to give the title compound as a white solid. Yield: 95%. mp 58–60 °C. ^1^H-NMR (CDCl_3_) δ 2.18 (s, 3H), 3.58 (s, 3H), 4.99 (s, 2H), 5.31 (s, 2H), 6.65 (s, 1H) , 6.7 (s, 1H), 6.75 (d, *J =* 8.67 Hz, 1H), 6.82 (s, 1H), 6.99 (d, *J =* 5.16 Hz, 1H), 7.22 (m, 3H), 7.35 (s, 1H) , 7.37 (s, 1H), 7.45 (s, 1H), 7.64 (s, 1H), 8.02 (dd, *J =* 8.67, 2.46 Hz, 1H), 8.44 (d, *J =* 5.16 Hz, 1H), 8.52 (d, *J =* 2.46 Hz, 1H). ^13^C-NMR (CDCl_3_) *δ* 21.58, 39.88, 55.28, 67.96, 111.02, 111.22, 113.77, 115.57, 118.95, 120.17, 121.19, 121.73, 127.98, 128.05, 128.60, 128.71, 130.27, 132.85, 137.18, 137.43, 140.04, 140.99, 145.55, 150.00, 151.41, 155.21, 159.74, 164.16; positive ion ESI-MS *m/z* 510.1909 (M+Na)^+^.

*2-(3-(3-Methoxy-5-methylphenyl)-4-(2-(6-fluoropyridin-3-yl)pyridin-4-yl)-1H-pyrazole-1-yl)acetonitrile* (**5j**). Flash column chromatography was carried out using hexane/ethyl acetate, 1:1 (v/v) to give the title compound as a white solid. Yield: 81%. mp 64–66 °C. ^1^H-NMR (CDCl_3_) δ 2.19 (s, 3H), 3.61 (s, 3H), 5.12 (s, 2H), 6.67 (s, 1H), 6.7 (s, 1H), 6.82 (s, 1H), 6.9 (dd, *J =* 8.55, 2.49 Hz, 1H), 7.09 (d, *J =* 5.08 Hz, 1H), 7.49 (s, 1H), 7.79 (s, 1H), 7.98 (dt, *J =* 8.55,2.49 Hz, 1H), 8.48 (s, 1H), 8.49 (s, 1H). ^13^C-NMR (CDCl_3_) δ 21.52, 39.97, 55.27, 109.32, 109.82, 111.21, 113.76, 115.43, 119.41, 119.83, 121.73, 130.33, 133.05, 140.08, 141.22, 146.00, 146.20, 150.20, 151.49, 153.84, 159.75, 162.38, 165.57; positive ion ESI-MS *m/z* 422.1386 (M+Na)^+^.

### 3.2. In Vitro Anticancer Screening

Cell line screening was performed at the National Cancer Institute (NCI, Bethesda, MD, USA) applying the following procedure: the human tumor cell lines of the cancer screening panel are grown in RPMI 1640 medium containing 5% fetal bovine serum and 2 mM L-glutamine. For a typical screening experiment, cells are inoculated into 96 well microtiter plates in 100 µL at plating densities ranging from 5000 to 40,000 cells/well depending on the doubling time of individual cell lines. After cell inoculation, the microtiter plates are incubated at 37 °C, 5% CO_2_, 95% air, and 100% relative humidity for 24 h prior to addition of experimental drugs. After 24 h, two plates of each cell line are fixed* in situ* with TCA, to represent a measurement of the cell population for each cell line at the time of drug addition (Tz).

Experimental drugs are solubilized in dimethyl sulfoxide at 400-fold the desired final maximum test concentration and stored frozen prior to use. At the time of drug addition, an aliquot of frozen concentrate is thawed and diluted to twice the desired final maximum test concentration with complete medium containing 50 µg/mL gentamicin. Additional four, 10-fold or ½log serial dilutions are made to provide a total of five drug concentrations plus control. Aliquots of 100 µL of these different drug dilutions are added to the appropriate microtiter wells already containing 100 µL of medium, resulting in the required final drug concentrations. Following drug addition, the plates are incubated for an additional 48 h at 37 °C, 5% CO_2_, 95% air, and 100% relative humidity. For adherent cells, the assay is terminated by the addition of cold TCA. Cells are fixed* in situ* by the gentle addition of 50 µL of cold 50% (w/v) TCA (final concentration, 10% TCA) and incubated for 60 min at 4 °C. The supernatant is discarded, and the plates are washed five times with tap water and air dried. Sulforhodamine B (SRB) solution (100 µL) at 0.4% (w/v) in 1% acetic acid is added to each well, and plates are incubated for 10 min at room temperature. After staining, unbound dye is removed by washing five times with 1% acetic acid and the plates are air dried. Bound stain is subsequently solubilized with 10 mM Trizma base, and the absorbance is read on an automated plate reader at a wavelength of 515 nm. For suspension cells, the methodology is the same except that the assay is terminated by fixing settled cells at the bottom of the wells by gently adding 50 µL of 80% TCA (final concentration, 16% TCA). Using the seven absorbance measurements [time zero, (Tz), control growth, (C), and test growth in the presence of drug at the five concentration levels (Ti)], the percentage growth is calculated at each of the drug concentrations levels. Percentage growth inhibition is calculated as:

[(Ti − Tz)/(C − Tz)] × 100 for concentrations for which Ti ≥ Tz.
[(Ti − Tz)/Tz] × 100 for concentrations for which Ti < Tz.

Three dose response parameters are calculated for each experimental agent. Growth inhibition of 50% (IC_50_) is calculated from [(Ti − Tz)/(C − Tz)] × 100 = 50, which is the drug concentration resulting in a 50% reduction in the net protein increase (as measured by SRB staining) in control cells during the drug incubation. The drug concentration resulting in total growth inhibition (TGI) is calculated from Ti = Tz. The LC_50_ (concentration of drug resulting in a 50% reduction in the measured protein at the end of the drug treatment as compared to that at the beginning) indicating a net loss of cells following treatment is calculated from [(Ti − Tz)/Tz] × 100 = −50. Values are calculated for each of these three parameters if the level of activity is reached; however, if the effect is not reached or is exceeded, the value for that parameter is expressed as greater or less than the maximum or minimum concentration tested.

### 3.3. DNA Topoisomerase IIα Inhibition Screening in Vitro

The DNA topoisomerase IIα inhibitory activity of the compounds was measured as follows [20]: a mixture containing 200 ng of supercoiled pBR322 plasmid DNA and 1 unit of human DNA topoisomerase IIα (USB Corp., Cleverland, OH, USA) was incubated with and without the prepared compounds in the assay buffer (10 mM Tris HCl (pH 7.9) containing 50 mM NaCl, 50 mM KCl, 5 mM MgCl_2_, 1 mM EDTA, 1 mM ATP, and 15 mg/mL bovine serum albumin) for 30 min at 30 °C. The reaction in a final volume of 20 mL was quenched by adding 3 mL of 7 mM EDTA. The reaction products were analyzed on 1% agarose gel at 25 V for 4 h with TAE as the running buffer. Gels were stained for 30 min in an aqueous solution of ethidium bromide (0.5 μg/mL). DNA bands were visualized by transillumination with UV light and supercoiled DNA was quantitated using AlphaImager^TM^ (Alpha Innotech Corporation, San Leandro, CA, USA).

## 4. Conclusions

The preliminary screening of this new set of compounds shed light on the effect of substitution on the distal pyridinyl moiety. Compounds **5c** and **5h** revealed a broad spectrum of activity while **5e** showed a high degree of selectivity toward several NCI cancerous cell lines. On that basis, we could say that **5c** and **5h** react with common cancer-related macromolecules while **5e** acts more specifically on some macromolecule responsible for driving the tumorigenicity of the SR leukemia cell line. COMPARE analysis has suggested one of these macromolecules is topoisomerase IIα enzyme, but on applying the* in vitro* screening assay for topoisomerase IIα enzyme, **5e** and **5i** have not shown any topoisomease IIα activity. In sum, these compounds still have an unknown mechanism of action and finding out the molecular target for this activity will be our next challenge.
